# The Role of *Dihydropyrimidine Dehydrogenase* and *Thymidylate Synthase* Polymorphisms in Fluoropyrimidine-Based Cancer Chemotherapy in an Iranian Population

**Published:** 2020

**Authors:** Mohammad Hadi Abbasian, Nafiseh Ansarinejad, Bahareh Abbasi, Masoud Iravani, Tayeb Ramim, Fahime Hamedi, Ali M. Ardekani

**Affiliations:** 1. Department of Medical Biotechnology, National Institute of Genetic Engineering and Biotechnology (NIGEB), Tehran, Iran; 2. Department of Hematology and Oncology, Hazrat Rasool-e Akram Hospital, Iran University of Medical Sciences, Tehran, Iran; 3. Cancer Pharmacogenetics Research Group (CPGRG), Iran University of Medical Sciences, Tehran, Iran; 4. Department of Medical Genetic, Medical Biotechnology Ins., National Institute of Genetic Engineering and Biotechnology (NIGEB), Tehran, Iran; 5. Tehran Gastroenterology and Hepatology Center, Tehran, Iran; 6. Department of Medicine, Faculty of Medicine, Tehran University of Medical Sciences, Tehran, Iran; 7. Department of Cell and Molecular Biology, Faculty of Biological Sciences, Kharazmi University, Tehran, Iran

**Keywords:** 5-fluorouracil, Dihydropyrimidine dehydrogenase, Fluoropyrimidines, Pharmacogenetics, Thymidylate synthase

## Abstract

**Background:**

The fluoropyrimidine drug 5-Fluorouracil (5-FU) and the prodrug capecitabine have been extensively used for treatment of many types of cancer including colorectal, gastric, head and neck. Approximately, 10 to 25% of patients suffer from severe fluoropyrimidine-induced toxicity. This may lead to dose reduction and treatment discontinuation. Pharmacogenetics research could be useful for the identification of predictive markers in chemotherapy treatment. The aim of the study was to investigate the role of five genetic polymorphisms within two genes (*DPYD, TYMS*) in toxicity and efficacy of fluoropyrimidine-based chemotherapy.

**Methods:**

Total genomic DNA was extracted from 83 cancer patients treated with fluoropyrimidine-based chemotherapy. In this study, three polymorphisms were genotyped in *dihydropyrimidine dehydrogenase* gene c.1905+1 G>A (DPYD*2A; rs3918290), c.1679 T>G (I560S; DPYD*13; rs55886062), and c.2846A>T (D949V; rs67376798) and two polymorphisms, besides the Variable Number of Tandem Repeat (VNTR) polymorphism and 6-*bp* insertion/deletion polymorphism in *thymidylate synthase* gene. The analysis of polymorphisms for rs3918290, rs55886062, rs67376798 and 6-*bp* insertion/deletion in TYMS was done by Polymerase Chain Reaction-restriction Fragment Length Polymorphism (PCRRFLP) TYMS VNTR analysis. 5-FU-related toxicities such as anemia, febrile neutropenia, neurotoxicity, vomiting, nausea, and mucositis were evaluated according to NCI-CTC criteria version 4.0. T-test and chi-square were used and p-values less than 0.05 were considered statistically significant.

**Results:**

*DPYD* gene polymorphisms were not observed in this study. The frequency of the TYMS +6 *bp* allele was 40.35% and the −6 *bp* allele was 59.65% in this study. The frequency of VNTR 2R allele was 48.75% and 3R allele was 51.15%. Toxicity grade II diarrhea, mucositis, nausea, vomiting, and neurotoxicity was 2.2, 24.1, 15.7, 6, and 51.8%, respectively. Thymidylate synthase ins/del polymorphisms were associated with increased grade III neurotoxicity (p=0.02). Furthermore, anemia grade III was significantly associated with 2R/2R genotype (0.009).

**Conclusion:**

*Thymidylate synthase* gene polymorphisms may play a key role in fluoropyrimidne -based chemotherapy. Although rare *DPYD* polymorphisms were not observed in our study, according to large population studies, *DPYD* gene polymorphisms could be used as a predictive biomarker for patient treatments.

## Introduction

The fluoropyrimidine drug 5-Fluorouracil (5-FU) and the prodrug capecitabine are used for treatment of a variety of solid cancers including gastrointestinal tract and breast ^1^.

5-FU/leucovorin combined with oxaliplatin (FOLFOX) is currently a standard chemotherapy regimen in treating colorectal and gastric cancers and has been shown clearly to improve response to treatment ^2,3^. However, the development of gastrointestinal, hematological and neurological toxicities is a major clinical problem following FOLFOX treatment ^4^. The clinical utility of DPYD polymorphisms has been demonstrated in a panel of markers to predict clinically actionable 5-FU toxicity. Other polymorphisms have been suggested as markers in TYMS, Enolase Superfamily Member 1 (ENOSF1) ^5^, MethyleneTetraHydroFolate Reductase (MTHFR) ^6^, ATP-binding cassette sub-family B member 1 (ABCB1) ^7^, and Cytidine Deaminase (CDA) ^8^.

Approximately, 85% of administered 5-FU dose is degraded via dihydropyrimidine dehydrogenase (DPD, encoded by the *DPYD* gene) ^9^. Functional Single-Nucleotide Polymorphisms (SNPs) in the *DPYD* gene alter DPD activity which may lead to the development of severe 5-FU related toxicities. The clinically most relevant variant is DPYD*2A (c.1905+1G>A, previously named IVS14+1G>A or DPD*2A), or rs3918290 ^10–13^; DPYD*13 (c.1679T>G(rs55886062) and c.2846 A>T (rs67376798) also result in low DPD activity and/or 5-fluorouracil toxicity ^12–17^.

A Variable Number of 28 *bp* Tandem Repeat (VNTR) within the promoter enhancer region (TSER) of TYMS (*Thymidylate Synthase* gene) is usually presented as a double-tandem repeat (2R) or a triple-tandem repeat (3R) ^18–20^. Second polymorphism is a 6-*bp* insertion/deletion in the 3′-untranslated region (3′-UTR) of TYMS ^21^. These two polymorphisms have been associated with altered TYMS expression, toxicity and an improved clinical response ^13,22,23^.

The aim of this study was to determine whether the five genetic polymorphisms within two genes (*DPYD, TYMS*) are associated with severe toxicity in patients with cancer receiving fluoropyrimidine-based chemotherapy.

## Materials and Methods

### Patients and study design

Eighty three cancer patients received 5-FU-based chemotherapy at Hazrat-e Rasool hospital and Masoud clinic, Tehran, Iran between February 2014 and June 2016. The cancer types among the patients were distributed as follows: 28 colon (33.7%), 37 rectum and rectosigmoid (44.5%), and 18 stomach (21.6%). Patients were questioned about nausea and vomiting, mucositis, diarrhea, handfoot syndrome, and neutropenia at every cycle. Eligible patients were treated with FOLFOX4 (Oxaliplatin 85 *mg/m*^2^ (2 *hr* infusion on day 1), leucovorin (100 *mg/m*^2^ as 2 *hr* infusion on day 1), 5- fluorouracil bolus (400 *mg/m*^2^) and 22 *hr* infusion (600 *mg/m*^2^) on days 1 and 2 every 2 weeks for 6 months (12 cycles). Toxicity was evaluated according to Common Terminology Criteria for Adverse Events (CTCAE), version 4 ^24^. Neurotoxicity was evaluated according to the oxaliplatin-specific scale. The study was approved by the ethics committees (NIGEB) (940101-IV-503). All patients signed a written informed consent before entering the study.

### Genotyping

Total genomic DNA was extracted from 200 *μl* whole blood using the GeneAll^®^ kit (South Korea) and stored at −20°*C* until genotyping. The TYMS VNTR polymorphism was amplified by polymerase chain reaction with Taq DNA Polymerase 2×Master Mix RED (Amplicon, Denmark). Other polymorphisms were performed using a PCR–RFLP technique. RFLP analysis was performed with fast digest enzymes (Thermo Fisher Scientific, the USA). After restriction enzyme analysis, PCR fragments were visualized in a 2.5–3% agarose gel. The information of studied genetic variants is shown in table 1.

**Table 1.** Characteristics of studied DPYD and TYMS polymorphisms

**Gene**	**SNP in dbSNP**	**Location**	**Amino acid change**	**Nucleotide change**	**Primer**	**Annealing temperature**	**Restriction enzyme ^b^**	**Product size (*bp*)**
***DPYD***								
	rs3918290	Intron 4	Exon 14 skipping	1905þ1G 4A	F:ACTCAATATCTTTACTCTTTCATCAGGAC R:ACATTCACCAACTTATGCCAATTCT	60	HpyCH4	N: 190+54 M: 244+190+54
	rs67376798	Exon 22	D949V	2846 A4T	F: ACCACAGTTGATACACATTTCTTGA ^a^ R:GCTTGCTAAGTAATTCAGTGGC	59	BclI	N: TT: 113+23 M: 136+113+23
	rs55886062	Exon 13	I560S	1679 T4G	F:TCACCAATACCAATAAGTTACACTGAGA R:TTAATTCGGATGCTGTGTTGAAGTG	61	Bsp119I	N: 469,265 M: 724,469,265
***TYMS***								
	rs45445694	*TS 5*_-untranslated region *(5*_*-UTR*	28 *bp* repeat		F:CTAAGACTCTCAGCTGTGGCCCTG R:CCACAGGCATGGCGCGGC	64		2R: 276 3R:304
	rs16430	*TS 3*_-untranslated region *(3*_-*UTR )*	6 *bp* deletion	Deletion or insertion of TTAAAG	F:CAAATCTGAGGGAGCTGAGTAACA R:CAAAGCGTGGACGAATGCAGA	60	DraI	Ins/ins: 60,63 Ins/del: 123,63,60

a: Underlined capital letter labeled nucleotides show mismatch to complementary DNA.

b: F=forward; R=reverse; N=normal; M=mutant.

### Statistical analysis

Statistical analysis was done by SPSS software version 21. The quantitative analysis was performed by descriptive variables. T-test was used for comparison of different groups and chi-square was used for qualitative analysis. p-value less than 0.05 was considered statistically significant.

## Results

### Genotyping

**Figure 1.**
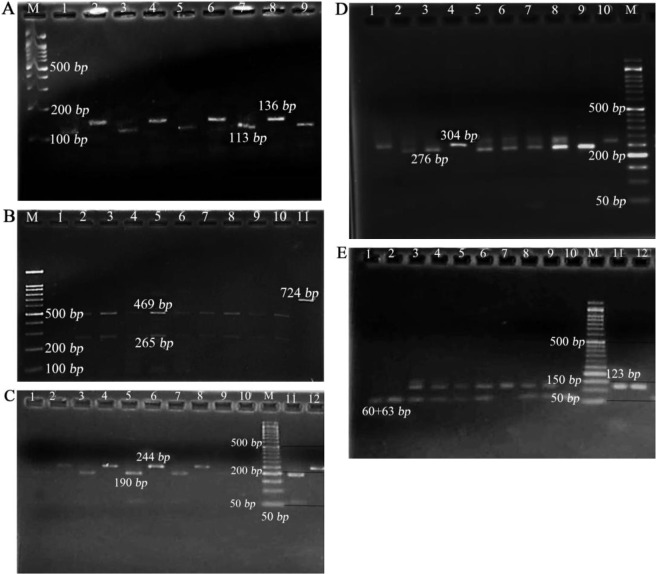
Polymorphism analysis of dihydropyrimidine dehydrogenase (DPYD) and thymidylate synthase (TYMS) polymorphism. A) rs 67376798; PCR product digested with BclI; lanes 1,3,5,7 and 9:113+23 *bp* (Wild type); lanes 2, 4, 6 and 8:136 *bp* (Undigested control); marker 100 *bp* (SinaClon, Tehran, Iran). B) rs55886062; PCR product digested with Bsp119I; lanes 1–10: 469+265 *bp* (Wild type); lanes 11:724 *bp* (Undigested control) marker 100 *bp* (Sina-Clon, Tehran, Iran). C) rs 3918290; product digested with Hpy CH4; lanes 1,3,5,7,9,11: 190+54 *bp* (Wild type); lanes 2,4,6,8,10,12: 244 *bp* (Undigested control); marker 50 *bp* (SinaClon, Tehran, Iran). D) rs45445694; lanes 3,9: 276 *bp* (2 repeat of 28 *bp* VNTR,2R); lanes 1,4,10: 304 *bp* (3 repeat of 28 *bp* VNTR,3R); lanes 2,5,6,7,8: 276+ 304 (2R3R); marker 50 *bp* (SinaClon, Tehran, Iran). E) TYMS 1494-del TTAAAG; lanes 1,2,10: 60+63 *bp* (6 *bp* deletion, del/del); lanes 7,11,12: 123 *bp* (6 *bp* insertion, ins/ins) lanes 3–6,8,9: 60+63+123 (ins/del); marker 50 *bp* (SinaClon, Tehran, Iran).

Eighty three cancer patients (58 males, 25 females, and mean age of 57.17 years old, range of 23–86 years) were evaluated in this study. Three variants in *DPYD* gene were not polymorphic in this study (Table 2, Figure 1). The distribution of the rs45445694 genotype was 27.7% for 2R/2R, 30.1% for 2R/3R and 42% for 3R/3R. The distribution of the rs16430 was 38.5% for del6/del6, 42.1% for del6/ins6 and 19% for ins6/ins6 (Table 3).

**Table 2.** Genotype frequencies of DPYD rs3918290, rs67376798, rs55886062 polymorphism

**Reference**	**Sample type**	**rs3918290 n (%)**			**rs67376798 n (%)**			**rs55886062 n (%)**		

		**GG**	**GA**	**AA**	**TT**	**TA**	**AA**	**AA**	**AC**	**CC**
**Our study**	Colorectal and gastric cancers	83 (100)	0 (0.0)	0 (0.0)	83 (100)	0 (0.0)	0 (0.0)	83 (100)	0 (0.0)	0 (0.0)
**(25)**	Colorectal cancer	99 (99)	1 (1)	0 (0.0)	100 (100)	0 (0.0)	0 (0.0)	99 (99)	1 (1)	0 (0.0)
**(23)**	Colorectal and oesophageal, anal and hepatobiliary cancers	427 (99)	3 (0.006)	0 (0.0)	426 (99)	4 (0.9)	0 (0.0)	429 (99)	1 (0.2)	0 (0.0)
**(10)**	Colon cancer	2859 (99)	27 (0.94)	0 (0.0)	2882 (99.8)	4 (0.14)	0 (0.0)	2854 (99.9)	32 (1.1)	0 (0.0)
**(13)**	Colon cancer, pancreatic, gastric, bile duct, esophageal tumors and breast cancer	670 (98)	13 (1.9)	0 (0.0)	-	-	-	-	-	-
**(26)**	Rectal cancer	131 (100)	0 (0.0)	0 (0.0)	-	-	-	-	-	-

**Table 3.** Genotype frequencies of TYMS rs45445694 and rs16430 polymorphisms

**Reference**	**Sample type**	**rs45445694 n (%)**			**rs16430 n (%)**		

		**2R/2R**	**2R/3R**	**3R/3R**	**del6/del6**	**del6/ins6**	**ins6/ins6**
**Our study**	Colorectal and gastric cancers	23 (27)	25 (31)	35 (42)	32 (38)	35 (42)	16 (19)
**(27)**	Colorectal cancer	61 (17)	156 (44)	119 (34)	32 (9)	154 (44)	155 (44)
**(28)**	Colorectal cancer	32 (19)	70 (43)	63 (38)	27 (16)	84 (51)	55 (35)
**(29)**	Colorectal cancer	14 (16)	44 (51)	28 (32)	6 (7)	45 (53)	34 (40)
**(30)**	Colorectal cancer	23 (17.6)	57 (43.8)	42 (32.3)	15 (11.5)	63 (48.4)	44 (33.8)

### Toxicity

Toxicity data included hematological toxicities (anemia and neutropenia) in 50 patients and nonhematologic toxicities (nausea, vomiting, diarrhea, mucositis, neurotoxicity) in 83 patients. In this study, 20% (17/83) developed non-hematological grade III–IV toxicity and 29% (13/44) developed hematological grade III–IV toxicity. Anemia (18%) was the most common severe hematological toxicity, whereas mucositis (3%) and neurotoxicity (2%) were the most frequent nonhematological severe toxicities. Also, 68% of patients experienced neurotoxicity as the most severe toxicity of any grade developed during the treatment. Thymidylate synthase ins/del polymorphisms were significantly associated with increased grade III neurotoxicity (p=0.02). Anemia grade III was significantly associated with 2R/2R genotype (0.009) (Table 4).

**Table 4.** Association between TYMS polymorphisms, rs16430 and rs3474303, and toxicity after FOLFOX chemotherapy

**Toxicity** ^a^	**Number of patients n (%)**	**rs16430**				**rs3474303**			

		**del6/del6**	**del6/ins6**	**ins6/ins6**	**p-value ^c^**	**2R2R**	**2R3R**	**3R3R**	**p-value**
**Vomiting grade II**	5 (6)	2 (40)	1 (20)	2 (40)	0.9	1 (20)	3 (60)	1 (20)	0.7
**Nausea grade II**	13 (15.7)	5 (38.4)	6 (46)	2 (15.3)		4 (30)	4 (30)	5 (38.4)	0.9
**Neurotoxicity grade II**	43 (51)	14 (32.5)	19 (44)	10 (23.2)	0.5	12 (27.9)	11 (25.5)	20 (46.5)	0.5
**Neurotoxicity grade III**	12 (14.5)	9 (75)	2 (16.6)	1 (8.3)	0.02 ^*^	5 (41.6)	4 (33.3)	3 (25)	0.3
**Neurotoxicity grade IV**	2 (2.4)	0 (0)	1 (50)	1 (50)	0.4	1 (50)	0 (0)	1 (50)	0.6
**Anemia grade ^b^III**	9 (10)	4 (33.3)	3 (44.4)	2 (22.2)	0.9	6 (66.7)	1 (11.1)	2 (22.2)	0.009 ^*^

a) Toxicity was evaluated according to Common Terminology Criteria for Adverse Events (CTCAE). Neurotoxicity was evaluated according to the oxaliplatin-specific scale.

b) 50 patients were evaluable for anemia.

c) Chi-square was performed for analyzing association between TYMS polymorphisms, rs16430 and rs3474303, and toxicity. p-values less than 0.05 are statistically significant.

## Discussion

Colorectal cancer is the second most common cancer in women and the third most common cancer in men worldwide ^31^. Colorectal cancer incidence is increasing in Iran, China and South Korea ^32,33^. Fluorouracil in combination with leucovorin, and oxaliplatin (FOLFOX) or with irinotecan (FOLFIRI) is the backbone of chemotherapy treatment for colorectal and gastric cancers ^28,34,35^. Gene polymorphisms are associated with cancer susceptibility and response to treatment in different types of cancer ^36^.

The aims of this study were to investigate the association of *DPYD* rare risk variants and *TYMS* polymorphisms and toxicity that may help predict the response to fluorouracil-based chemotherapy. These polymorphisms were evaluated in 83 colorectal and gastric cancer patients treated with FOLFOX regimen.

Dihydropyrimidine Dehydrogenase (DPD) is responsible for conversion of 5-FU to 5-fluorodihydrouracil (5-FUH2) and plays a key role in the catabolism of 5-FU. Deficiency in DPD activity leads to severe toxicity which may lead to treatment discontinuation or even death. Three *DPYD* variants of rs3918290, rs- 55886062 and rs67376798 were associated with decreased DPD enzyme activity and a high risk of severe 5-FU toxicity ^5,37–39^. The development of severe toxicity results in dose reduction, discontinuation of treatment or even death in cancer patients ^40–43^.

Regarding the low minor allele frequency of the *DPYD* rare risk variants, three *DPYD* variants of rs-3918290, rs55886062 and rs67376798 were not identified in the study group. This finding is consistent with some small studies ^26^. Similar to our study, rs3918290 polymorphism was not found in Korean, Taiwanese, Turkish, Caucasian, and African populations ^44–47^. In some large studies, heterozygous patients for the rs-3918290 experienced severe toxicity ^23,48^. Some reported toxicity in just 46% of patients with rs3918290 polymorphism ^13^. For the rs67376798 variant, previous studies reported that 75% of patients with a heterozygous genotype experienced severe toxicity in the first two cycles of therapy ^23^. Other studies showed 60% or 62% of heterozygous patients experiencing toxicity within the first two cycles ^13,48^. The single patient with a heterozygous rs55886062 genotype experienced late toxicity after cycle 2. Loganayagam *et al* reported, in the largest study to date, the incidence of grade ≥3 5FU-toxicities in rs3918290, rs55886062, and rs-67376798 carriers as 88.0, 50.0, and 81.5%, respectively, whereas incidence of grade≥3 overall toxicities was 88.0, 75.0, and 88.9%, respectively. Carrier of rs-3918290, rs55886062 and rs37376798 had at least one grade IV toxicity of 64.0, 25.0 and 66.7%, respectively ^10^.

The Clinical Pharmacogenetics Implementation Consortium (CPIC) recommended reduction of fluoropyrimidine dosage in patients who are heterozygous for rs3918290, rs55886062 and rs67376798 variants which may prevent severe and possibly life-threatening toxicities. The use of 5-fluorouracil or capecitabine is not recommended in patients who are homozygous for rs3918290, rs55886062 and rs67376798 ^16^.

Thymidylate synthase is a main intracellular target of the active 5-FU metabolite, FdUMP, which forms a ternary complex with TS and 5, 10-MTHF. rs3474303 as a variable number of tandem repeats (VNTR) is present in the 5′-untranslated region (5′-UTR) of the *TS* gene (*TYMS*). The three 28-*bp* repeat (3R) is associated with 3–4-fold translational efficiency, compared to two 28-*bp* repeat. In our study, the two 28-*bp* repeat and the 6-*bp* deletion allele frequencies of the *TYMS* gene were 48.75 and 59.65%, respectively. Significant associations were found between anemia grade III and 2R/2R TYMS genotype (0.009). In our study, association between polymorphism rs3474303 with toxicity is the same as other studies ^13,20,29,49–51^. 2R/3R or 3R/3R genotypes were significantly associated with a lower risk of toxicity. Although some studies have shown association between rs3474303 ^52^ and toxicity, this association is likely to be small and clinical test would not be useful ^5^.

The 6-*bp* deletion in the 3′-UTR region of the *TYMS* gene affects TYMS mRNA expression ^21^. In our study, the del/del genotype in rs16430 was significantly associated with increased grade III neurotoxicity (p=0.02). Other studies have shown del/del genotype in rs16430 associated with diarrhea, neutropenia and mucositis (p=0.0123) ^23^. However, certain other studies have failed to show an association between a homozygous del/del genotype and severe toxicity ^53,54^.

TS mRNA level predicts fluoropyrimidine and raltitrexed sensitivity in gastric cancer ^55^. A meta-analysis has shown that response rates for fluoropyrimidine-based chemotherapy were significantly lower in gastric cancer patients with high TS expression ^56^. In Meulendijks *et al*’s study, *TYMS* VNTR 3 R/3 R genotype was formally associated with Objective Response Rate (ORR) ^57^.

Homozygous genotype rs1801159A/A was associated with response to fluorouracil-based adjuvant chemotherapy in gastric cancer patients ^58^. Another study revealed that gene polymorphisms of *DPYD* could be considered as a biomarker for prediction of gastric cancer patients survival treated with 5-fluorouracil-based adjuvant chemotherapy ^59^.

## Conclusion

In conclusion, an association was found between VNTR and *TYMS* 1494 ins/del polymorphism in *TYMS* gene and anemia and neurotoxicity, respectively. Due to the relatively limited sample size, DPYD rare risk variants of rs3918290, rs55886062 and rs67376798 were not identified in our study. Further investigations on larger sample size are needed to demonstrate the role of rare risk variants of *DPYD* gene. In addition, to achieve better biomarkers for FOLFOX chemotherapy regimen, additional variation in genes involved in metabolism of other drugs like oxaliplatin should be investigated.
